# Accuracy of four mononucleotide-repeat markers for the identification of DNA mismatch-repair deficiency in solid tumors

**DOI:** 10.1186/s12967-017-1376-4

**Published:** 2018-01-12

**Authors:** Yuko Takehara, Takeshi Nagasaka, Akihiro Nyuya, Tomoko Haruma, Junko Haraga, Yoshiko Mori, Keiichiro Nakamura, Toshiyoshi Fujiwara, C. Richard Boland, Ajay Goel

**Affiliations:** 1 Department of Gastroenterological Surgery, Okayama University Graduate School of Medicine, Dentistry and Pharmaceutical Sciences, Okayama, Okayama 700-8558 Japan; 2 Department of Clinical Oncology, Kawasaki Medical School, 577 Matsushima, Kurashiki-City, Okayama 701-0192 Japan; 3 Department of Obstetrics and Gynecology, Okayama University Graduate School of Medicine, Dentistry and Pharmaceutical Sciences, Okayama, Okayama 700-8558 Japan; 4 Center for Gastrointestinal Research, Center for Translational Genomics and Oncology, Baylor Scott& White Research Institute and Charles A Sammons Cancer Center, Baylor University Medical Center, 3410 Worth Street, Suite 610, Dallas, TX 75246 USA

**Keywords:** Colorectal cancer, Endometrial cancer, Microsatellite instability, DNA mismatch repair, Hypermutated tumors

## Abstract

**Background:**

To screen tumors with microsatellite instability (MSI) arising due to DNA mismatch repair deficiency (dMMR), a panel of five quasi-monomorphic mononucleotide-repeat markers amplified in a multiplex PCR (Pentaplex) are commonly used. In spite of its several strengths, the pentaplex assay is not robust at detecting the loss of MSH6-deficiency (dMSH6). In order to overcome this challenge, we designed this study to develop and optimize a panel of four quasi-monomorphic mononucleotide-repeat markers (Tetraplex) for identifying solid tumors with dMMR, especially dMSH6.

**Methods:**

To improve the sensitivity for tumors with dMMR, we established a quasi-monomorphic variant range (QMVR) of 3–4 bp for the four Tetraplex markers. Thereafter, to confirm the accuracy of this assay, we examined 317 colorectal cancer (CRC) specimens, comprising of 105 dMMR [45 MutL homolog (MLH)1-deficient, 45 MutS protein homolog (MSH)2-deficient, and 15 MSH6-deficient tumors] and 212 MMR-proficient (pMMR) tumors as a test set. In addition, we analyzed a cohort of 138 endometrial cancers (EC) by immunohistochemistry to determine MMR protein expression and validation of our new MSI assay.

**Results:**

Using the criteria of ≥ 1 unstable markers as MSI-positive tumor, our assay resulted in a sensitivity of 97.1% [95% confidence interval (CI) = 91.9–99.0%] for dMMR, and a specificity of 95.3% (95% CI = 91.5–97.4%) for pMMR CRC specimens. Among the 138 EC specimens, 41 were dMMR according to immunohistochemistry. Herein, our Tetraplex assay detected dMMR tumors with a sensitivity of 92.7% (95% CI = 80.6–97.5%) and a specificity of 97.9% (95% CI = 92.8–99.4%) for pMMR tumors. With respect to tumors with dMSH6, in the CRC-validation set, Tetraplex detected dMSH6 tumors with a sensitivity of 86.7% (13 of 15 dMSH6 CRCs), which was subsequently validated in the EC test set as well (sensitivity, 75.0%; 6 of 8 dMSH6 ECs).

**Conclusions:**

Our newly optimized Tetraplex system will help offer a robust and highly sensitive assay for the identification of dMMR in solid tumors.

**Electronic supplementary material:**

The online version of this article (10.1186/s12967-017-1376-4) contains supplementary material, which is available to authorized users.

## Background

Microsatellite instability (MSI) is characterized by the accumulation of insertion-deletion mutations at microsatellite-repeat sequences and represents a hallmark feature of cancer cells with DNA mismatch-repair deficiency (dMMR) [1, 2]. Inactivation of any one or a combination of MMR genes, including *MutL homolog (MLH)1*, *MutS protein homolog (MSH)2*, *MSH6*, and *PMS2*, can result in MSI. Originally, MSI was discovered to correlate with germline defects in MMR genes in patients with Lynch syndrome, where > 90% of colorectal cancer (CRC) patients exhibit this phenotype [3, 4]. It was later recognized that MSI also occurs in ~ 12 to ~ 15% of sporadic CRCs that lack germline MMR mutations; however, in these patients, MSI manifests due to methylation-induced silencing of the *MLH1* promoter [5, 6]. Determination of MMR deficiency by MSI status or immunohistochemical staining for MMR proteins in CRC patients has clinical significance due to its prognostic and therapeutic implications [7]. Patients with MSI CRCs typically have better prognosis, although these cancers are less responsive to 5FU-based adjuvant chemotherapy [8]. Recently, clinical trials demonstrated the utility of MSI status in predicting response to PD-1 blockade in advanced unresectable solid tumor patients [9–11]. Additionally, MSI status was a significant predictor of the immune-related objective response rate [40% in dMMR CRC, 71% in dMMR non-CRC, 0% in MMR-proficient (pMMR) CRC] and immune-related progression-free survival rates (78, 67, and 11%, respectively) [9].

The procedures and criteria used to determine MSI in tumors are constantly evolving; however, there remains a lack of consensus regarding the most practical and robust MSI assay allowing for inexpensive clinical use and capable of providing consistent and reproducible results in laboratories worldwide [12]. In an effort to unify MSI analysis in CRC patients, in 1997, a National Cancer Institute (NCI) workshop recommended the use of a reference panel of five markers: two mononucleotide-repeat markers (BAT26 and BAT25) and three dinucleotide-repeat markers (D2S123, D5S346, and D17S250) [13]. In a follow-up NCI workshop, the panel recognized that the original markers had limitations, primarily due to the inclusion of the three dinucleotide-repeat markers [14]. First, it was noted that the dinucleotide-repeat markers were more suitable for identifying MSI-low tumors, whereas mononucleotide-repeat markers were more specific and sensitive for the determination of MSI-positive CRCs [15]. Second, due to the polymorphic nature of dinucleotide markers, these required PCR amplification of both the tumor DNA and matching normal specimens from each individual in order to interpret the results. Third, the conventional NCI-panel markers inadequately identified MSH6-deficient CRCs. Employing a panel of five quasi-monomorphic mononucleotide-repeat markers in a pentaplex PCR obviated the need for obtaining normal DNA from each CRC patient and offered better specificity and sensitivity relative to the NCI-panel markers [16]. Unfortunately, despite its obvious strengths, the pentaplex MSI approach gained limited acceptance for MSI-based screening of CRC patients, possibly due to a lack of clear understanding of the technical aspects of the assay and a paucity of data enabling its validation in independent laboratories. To address this concern, we previously performed a comprehensive determination of the accuracy of the pentaplex-panel markers in a large series of dMMR and pMMR CRCs by analyzing the PCR-amplified profiles of each marker in both tumor and matching normal DNA [17]. Based on the results of that study, we found that a smaller panel of only three markers, BAT26, NR21, and NR27, were adequate or better in identifying dMMR CRCs as compared with the original panel of five mononucleotide markers [17]. However, despite various practical and technical strengths of this panel of MSI markers for identifying MSI-positive CRCs, one of the limitations of these markers was their lack of robustness in identifying CRCs exhibiting MSH6-deficiency [17]. These data highlighted the need for developing a more robust assay capable of addressing this important issue and successfully identifying CRCs lacking MSH6.

Therefore, in the present study, we examined a panel of four quasi-monomorphic mononucleotide-repeat markers (BAT26, NR21, NR27, and CAT25) amplified in a single multiplex PCR reaction (Tetraplex) to determine its performance in detecting dMMR CRCs. This assay was first performed in a cohort of 318 CRC specimens, comprising 105 dMMR and 213 pMMR cases. Because the frequency of MSI tumor is the highest in the endometrium [2], we also analyzed the performance of our MSI assay in another cohort of 138 specimens with endometrial cancer (EC) exhibiting known MMR status.

## Methods

### CRC specimens in the test cohort

Matched germline and tumor DNA from 212 CRC patients diagnosed as pMMR by immunohistochemical (IHC) staining were collected from patients at the Okayama University Hospital (Okayama, Japan). Specimens from CRC patient with tumors diagnosed as dMMR (105) were also collected at three different institutions, including: (1) Baylor University Medical Center (Dallas, TX, USA); (2) University of Heidelberg (Heidelberg, Germany); and (3) Okayama University Hospital (Okayama, Japan). This cohort of 105 dMMR CRC tumors included 45 MLH1-deficient (dMLH1), 45 MSH2-deficient (dMSH2), and 15 MSH6-deficient (dMSH6) tumors. Tumor DNA was extracted from serial sections (5 μm) from the 105 formalin-fixed paraffin-embedded (FFPE) tumor tissues. FFPE samples were routinely stained, and representative tumor regions were identified for DNA extraction by microscopic examination. Genomic DNA was isolated from paraffin-embedded tissues using the QIAamp DNA mini kit (Qiagen, Valencia, CA). The Institutional Review Board at each of the three institutions granted approval for this study.

### EC specimens for the validation cohort

A total of 138 tumor samples were collected from EC patients at Okayama University Hospital (Okayama, Japan). Among these, 23 were dMLH1, eight were dMSH2, eight were dMSH6, and two were PMS2-deficient. Tumor DNA was collected and extracted as described for the CRC tumors. The Institutional Review Board at Okayama University Hospital granted approval for this study.

### MMR protein IHC

We examined MMR-protein expression for the MLH1, MSH2, MSH6, and PMS2 proteins in primary tumors from 317 CRC and 138 EC patients by IHC. Thin (5 µm) sections of representative blocks were deparaffinized and dehydrated using an ethanol gradient. Following antigen retrieval in citrate buffer (pH 6.0), endogenous peroxidase was blocked with 3% H_2_O_2_. Thereafter, slides were incubated overnight in the presence of purified mouse monoclonal antibodies against MLH1 (clone G168-15; 1:50; BD Pharmingen, San Diego, CA, USA), MSH2 (clone G219-1129; 1:200; BD Pharmingen), MSH6 (clone 44/MSH6; 1:100; BD Pharmingen), and PMS2 (clone A16-4; 1:200; BD Pharmingen). Additional incubations were performed with a biotin-conjugated secondary antibody (Vector Laboratories, Burlingame, CA, USA), the avidin–biotin–peroxidase complex (Vector Laboratories, Burlingame, CA, USA), and with biotinyl tyramide, followed by streptavidin peroxidase. Diaminobenzidine was used as a chromogen, and hematoxylin was used as a nuclear counterstain. Tumor cells were scored as negative for MMR-protein expression only if the epithelial cells within the tumor tissue lacked nuclear staining while the surrounding stromal cells were positive for MMR staining. Tumor tissue with all MMR proteins present were defined as pMMR, and those showing deficiency in at least one of the four MMR proteins were defined as dMMR.

### Tetraplex system and quasi-monomorphic variation range (QMVR) definition

MSI analysis was performed using four mononucleotide-repeat microsatellite targets (CAT25, NR21, NR27, and BAT26) in a single multiplex PCR reaction (Tetraplex). Primer sequences are shown in Additional file 1: Table S1, and each sense primer was end-labeled with one of the following fluorescent markers: PET, NED, VIC, or 6-FAM. PCR conditions for the Tetraplex assay consisted of an initial 15-min denaturation step at 95 °C, followed by 35 cycles at 95 °C for 30 s, 55 °C for 30 s, and 72 °C for 30 s, with a final extension at 72 °C for 10 min. Amplified PCR products were diluted with formamide and subjected to electrophoresis using an Applied Biosystems 310 Avant automated capillary electrophoresis DNA sequencer (Applied Biosystems, Foster City, CA, USA). Allelic sizes for each of the markers were determined using GeneMapper 3.1 software (Applied Biosystems).

Determination and validation of the QMVR for each of the four MSI markers was performed by individually scoring PCR-amplification profiles, and the size of both alleles was determined for each marker and for each tumor individually as described previously [17, 18].

### Statistical analyses

We used logistic regression analysis to examine the diagnostic performance of measuring MMR status in CRCs by utilizing different strategies to define MSI. Analyses were performed using JMP (v10.0.2; SAS Institute, Cary, NC, USA).

## Results

### Determination of QMVR for each marker by germline DNA

**Fig. 1:**
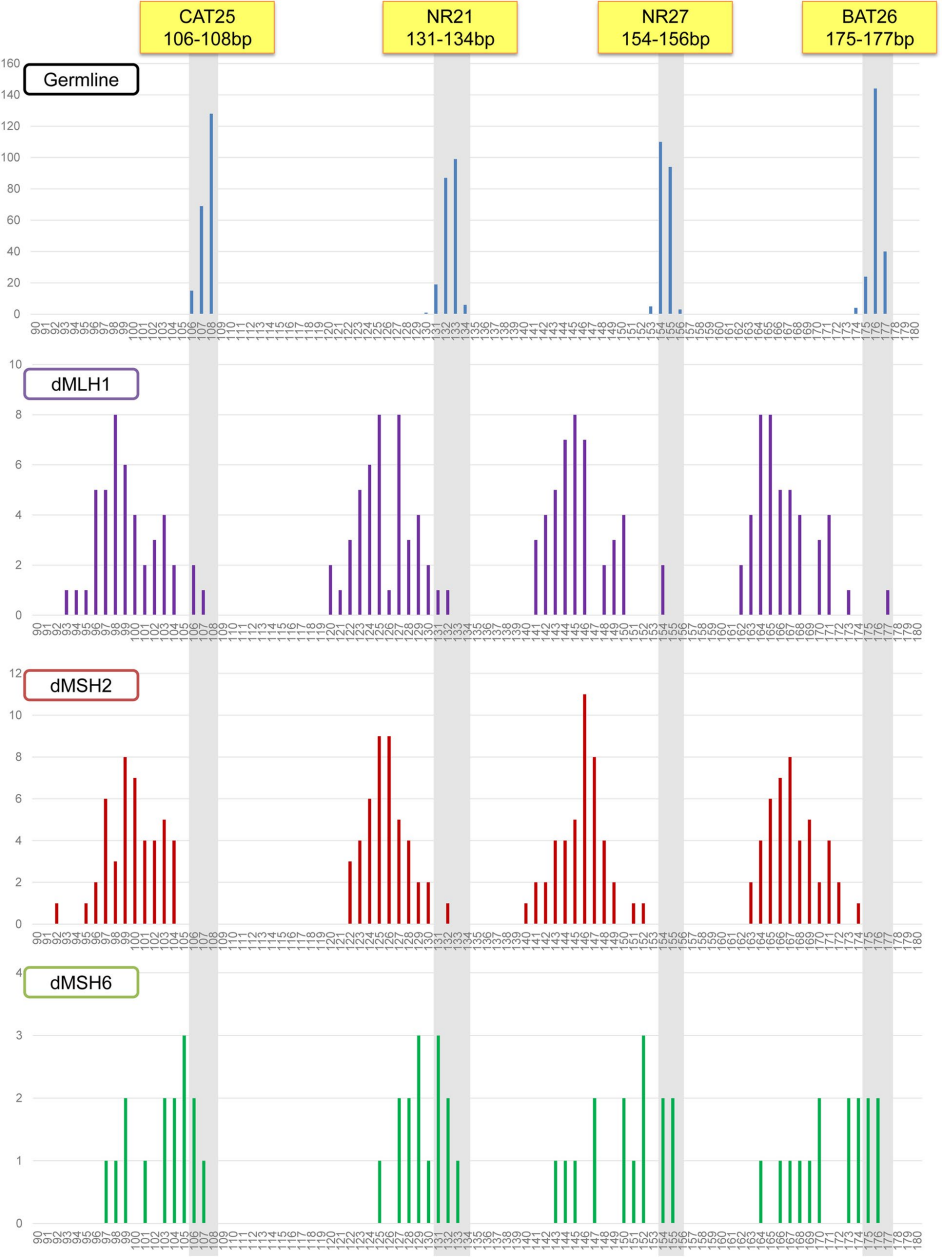
Frequency of allele-size distribution (in base pairs) for the four individual markers in the test set. Allele-size distribution from 212 pMMR tumors and their corresponding normal mucosa, 45 dMLH1, 45 dMSH2, and 15 dMSH6 tumors. For each marker, gray shading indicates the adjusted QMVR

Mono-nucleotide repeat markers are highly monomorphic in germline DNA from a wide spectrum of populations worldwide [18]. Theoretically, the QMVR for each marker should be constant in each experimental setting; however, results indicate that specific instrumentation or reagents might affect allele-size measurements for each marker [18]. This requires a one-time careful validation of QMVR in germline DNA prior to analysis of tumor MSI. We amplified matching germline DNA and tumor DNA from 212 CRC patients with pMMR tumors based on IHC staining in order to determine the QMVR for each MSI marker. Figure 1 shows the QMVR and sifted size of dMMR cases for each marker in the test set. The polymorphic range for each mononucleotide-repeat marker in germline DNA was as follows: CAT25 (106–108 bp), NR21 (131–134 bp), NR27 (153–156 bp), and BAT26 (175–177 bp). The most common allele for each of the markers was as follows: CAT25 (108 bp), NR21 (133 bp), NR27 (154 bp), and BAT26 (176 bp). As a comparison, the theoretical size of each marker is shown in Additional file 1: Table S1: CAT25 (109 bp), NR21 (133 bp), NR27 (159 bp), and BAT26 (182 bp) (a MSI case determined by Tetlaplex PCR assay is shown in Additional file 2: Figure S1). These results indicated that our amplification system reported the most common alleles as being shorter than the theoretical size for each marker (CAT25 by 1 bp, NR21 by 0 bp, NR27 by 5 bp, and BAT26 by 6 bp).

### Performance characteristics of *Individual* Allelic Markers for the identification of MMR-deficient CRCs in the test set

We examined the performance characteristics of individual markers for identifying CRCs with dMMR (Table 1). Our analysis clearly showed that all four mononucleotide-repeat markers were able to detect dMMR CRCs with a *sensitivity* that varied from 91.4% (NR21) to 95.2% (BAT26) and a *specificity* for pMMR CRCs from 97.6% (NR27) to 100% (CAT25). The highest sensitivity was observed with dMSH2 tumors, whereas the lowest sensitivity was observed with dMSH6 tumors.

**Table 1:** Performance characteristics of each MSI marker for the identification of MMR-deficient CRCs in the test set (317 CRCs)

Marker	References	Sensitivity% (95% CI)		Specificity% (95% CI) for pMMR	PPV% (95% CI) for dMMR	NPV% (95% CI) for pMMR
CAT25	QMVR (106–108 bp)	for dMMR (n = 105)	94.3 (88.1–97.4)	100.0 (98.2–100)	100 (96.3–100)	97.3 (94.1–98.7)
		dMLH1 (n = 45)	93.3 (82.1–97.7)			
		dMSH2 (n = 45)	100 (92.1–100)			
		dMSH6 (n = 15)	80.0 (54.8–93.0)			
NR21	QMVR (131–134 bp)	for dMMR (n = 105)	91.4 (84.5–95.4)	99.5 (97.4–99.9)	99.0 (94.3–99.8)	95.9 (92.4–97.8)
		dMLH1 (n = 45)	95.6 (85.2–98.8)			
		dMSH2 (n = 45)	97.8 (88.4–99.6)			
		dMSH6 (n = 15)	60.0 (35.7–80.2)			
NR27	QMVR (153–156 bp)	for dMMR (n = 105)	94.3 (88.1–97.4)	97.6 (94.6–99.0)	95.2 (89.2–97.9)	97.2 (94.0–98.7)
		dMLH1 (n = 45)	95.6 (85.2–98.8)			
		dMSH2 (n = 45)	100 (92.1–100)			
		dMSH6 (n = 15)	73.3 (48.0–89.1)			
BAT26	QMVR (175–177 bp)	for dMMR (n = 105)	95.2 (89.3–97.9)	98.1 (95.2–99.3)	96.2 (90.5–98.5)	97.7 (94.6–99.0)
		dMLH1 (n = 45)	97.8 (88.4–99.6)			
		dMSH2 (n = 45)	100 (92.1–100)			
		dMSH6 (n = 15)	73.3 (48.0–89.1)			

*PPV* positive predictive value, *NPV* negative predictive value

### Performance of the Tetraplex system for identification of dMMR tumors in the test set

We then examined the ability of the combination of all four allelic markers to determine dMMR or pMMR in CRCs using the Tetraplex system. When allelic-size variations at ≥ 1 of the four markers was defined as diagnosis of MSI, the Tetraplex system displayed 97.1% (95% confidence interval [CI] = 91.9–99.0%] sensitivity for identification of dMMR CRCs and 95.3% (95% CI = 91.5–97.4%) specificity for pMMR CRCs (Table 2).

**Table 2:** Performance characteristics of Tetraplex system with reference to QMVR for identification of MMR-deficient CRCs in the test set

No. of markers displaying allelic variation	The Tetraplex marker panel			
	Sensitivity (%)	Specificity (%)	PPV (%)	NPV (%)
4	88.6 (81.1–93.3)	100 (98.2–100)	100 (96.0–100)	94.6 (90.9–96.9)
3	95.2 (89.3–97.9)	100 (98.2–100)	100 (96.3–100)	97.7 (94.7–99.0)
2	96.2 (90.6–98.5)	100 (98.2–100)	100 (96.4–100)	98.1 (95.3–99.3)
1	97.1 (91.9–99.0)	95.3 (91.5–97.4)	91.1 (84.3–95.1)	98.6 (95.8–99.5)

Results are expressed as percentages (%), with 95% confidence intervals in parentheses

*PPV* positive predictive value, *NPV* negative predictive value

**Fig. 2:**
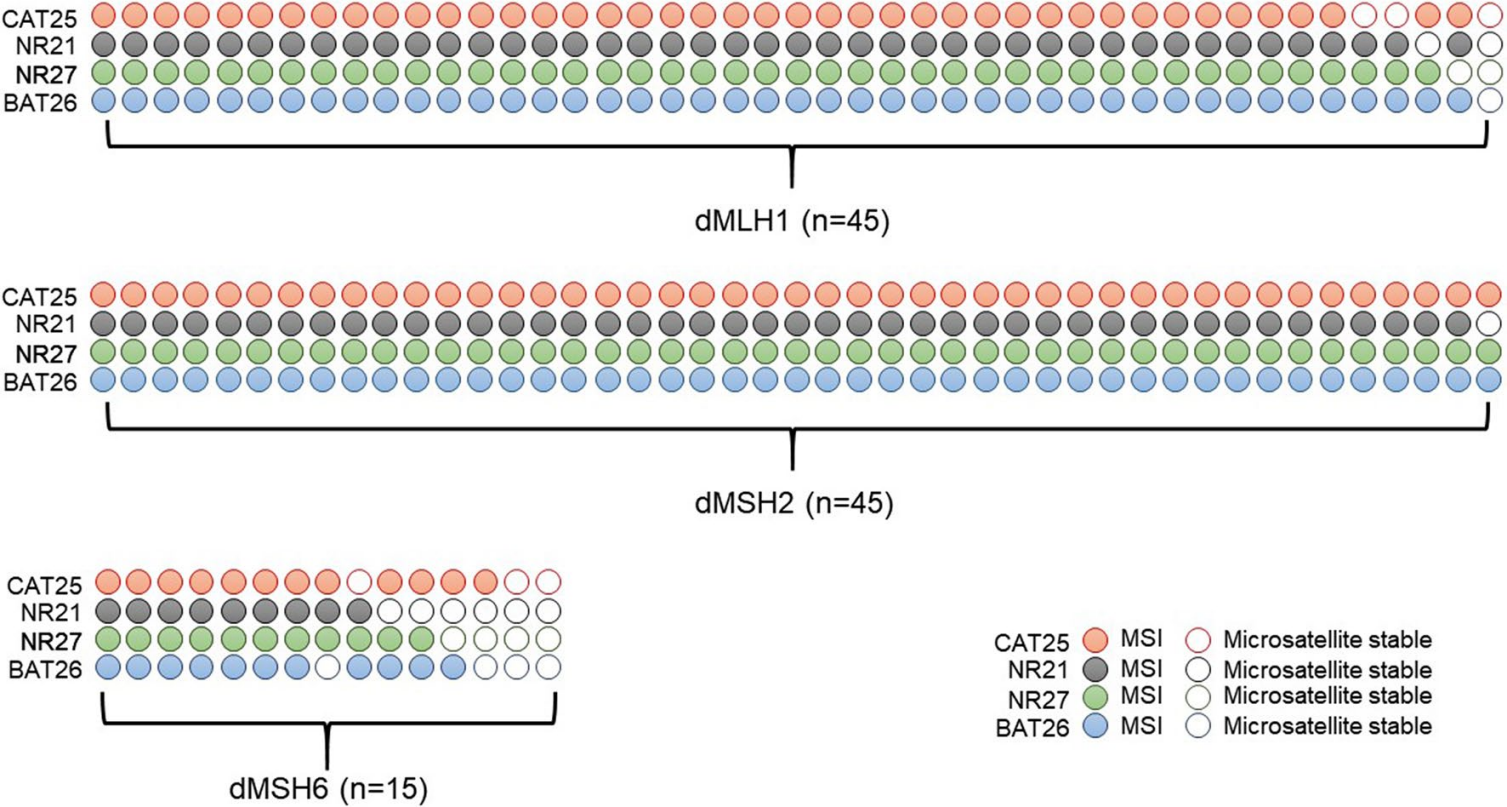
The performance of the Tetraplex system in the test set. A colored circle denotes a positive for allelic variation (or MSI), and an empty circle indicates a negative for variation (or microsatellite stable) in this specific allele

With respect to correlation of MSI data with MMR-protein-expression status, the Tetraplex system demonstrated a very high sensitivity for dMLH1 (100%) and dMSH2 tumors (100%). Additionally, this system was sufficiently robust for the detection of dMSH6 tumors with a sensitivity of 86.7% (13 of 15) when MSI was defined as instability at ≥ 1 of four markers (Fig. 2).

### Profiles of the MMR-expression Status of ECs and QMVR distribution for each marker in the validation set

**Fig. 3:**
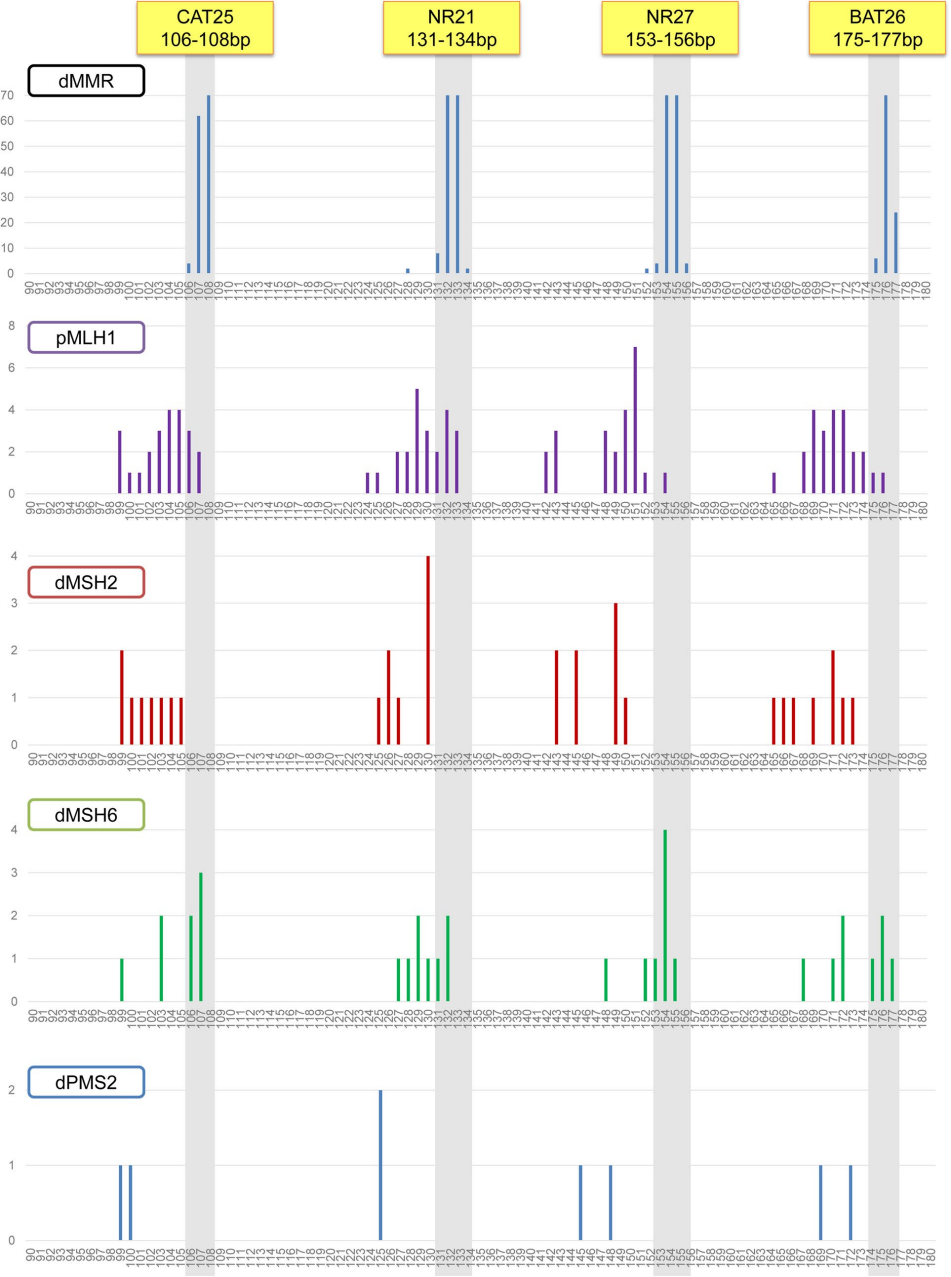
Frequency of allele-size distribution (in base pairs) for the four individual markers in the validation set. Allele-size distribution from 97 pMMR ECs: 23 dMLH1, eight dMSH2, eight dMSH6, and two PMS2-deficient ECs. For each marker, gray shading indicates the adjusted QMVR

To determine the ability of the Tetraplex system to detect dMMR tumors in other types of cancer, we performed similar analyses on EC specimens. We first examined the expression status of MMR proteins in a cohort of 138 ECs. IHC staining confirmed 41 ECs (29.7%) as dMMR: 23 ECs with dMLH1 (56.1% of ECs with dMMR), eight with dMSH2 (19.5%), eight with dMSH6 (19.5%), and two with PMS2 deficiency (4.9%). We then amplified the four allelic markers used for CRC and determined the distribution of QMVR and size-sorted alleles within each dMMR type. As shown in Fig. 3, there was a clear shift to smaller allele size in dMMR samples as compared to that observed in pMMR samples.

### Performance characteristics of *Individual* Allelic Markers for the identification of MMR-deficient ECs in the validation set

We then examined the performance characteristics of individual markers for identifying dMMR ECs (Table 3). Our analysis clearly showed the ability of the four mononucleotide-repeat markers to detect dMMR ECs. The sensitivity for dMMR in the different alleles varied from 78.1 to 90.2%, and the specificity for pMMR ECs was from 99.0 to 100%.

**Table 3:** Performance characteristics of each MSI marker for the identification of MMR-deficient ECs in validation test set (n = 138)

Marker	References	Sensitivity% (95% CI)		Specificity% (95% CI) for pMMR	PPV% (95%CI) for dMMR	NPV% (95% CI) for pMMR
CAT25	QMVR (106–108 bp)	for dMMR (n = 41)	80.5 (66.0–89.8)	100 (96.2–100)	100 (89.6–100)	92.4 (85.7–96.1)
		dMLH1 (n = 23)	82.6 (62.9–93.0)			
		dMSH2 (n = 8)	100 (67.5–100)			
		dMSH6 (n = 8)	50.0 (21.5–78.5)			
		dPMS2 (n = 2)	100 (34.2–100)			
NR21	QMVR (131–134 bp)	for dMMR (n = 41)	78.1 (63.3–88.0)	99.0 (94.4–99.8)	97.0 (84.7–99.5)	91.4 (84.5–95.4)
		dMLH1 (n = 23)	69.6 (49.1–84.4)			
		dMSH2 (n = 8)	100 (67.5–100)			
		dMSH6 (n = 8)	62.5 (30.6–86.3)			
		dPMS2 (n = 2)	100 (34.2–100)			
NR27	QMVR (153–156 bp)	for dMMR (n = 41)	90.2 (77.5–96.1)	99.0 (94.4–99.8)	97.4 (86.5–99.5)	96.0 (90.2–98.4)
		dMLH1 (n = 23)	95.7 (79.0–99.2)			
		dMSH2 (n = 8)	100 (67.5–100)			
		dMSH6 (n = 8)	62.5 (30.6–86.3)			
		dPMS2 (n = 2)	100 (34.2–100)			
BAT26	QMVR (175–177 bp)	for dMMR (n = 39)	87.8 (74.5–94.7)	100 (96.2–100)	100 (90.4–100)	95.1 (89.0–97.9)
		dMLH1 (n = 23)	91.3 (73.2–97.6)			
		dMSH2 (n = 8)	100 (67.5–100)			
		dMSH6 (n = 8)	62.5 (30.6–86.3)			
		dPMS2 (n = 2)	100 (34.2–100)			

*PPV*, positive predictive value, *NPV* negative predictive value

### Performance of the Tetraplex system for identification of dMMR tumors in the EC validation set

We examined the performance of all markers using the Tetraplex system in both dMMR and pMMR ECs. With allelic-size variations at ≥ 1 of the four markers defined as diagnosis of MSI, the Tetraplex system displayed 92.7% (95% CI = 80.6–97.5%) sensitivity for identification of dMMR ECs and 97.9% (95% CI = 92.8–99.4%) specificity for pMMR ECs (Table 4).

**Table 4:** Performance characteristics of Tetraplex system with reference to QMVR for identification of MMR-deficient ECs in the validation set (n = 138)

No. of markers displaying allelic variation	The Tetraplex marker panel			
	Sensitivity (%)	Specificity (%)	PPV (%)	NPV (%)
4	70.7 (55.5–82.4)	100 (96.2–100)	100 (88.3–100)	89.0 (81.7–93.6)
3	85.4 (71.6–93.1)	100 (96.2–100)	100 (90.1–100)	94.2 (87.9–97.3)
2	87.8 (74.5–94.7)	100 (96.2–100)	100 (90.4–100)	95.1 (89.0–97.9)
1	92.7 (80.6–97.5)	97.9 (92.8–99.4)	95.0 (83.5–98.6)	96.9 (91.4–99.0)

Results are expressed as percentages (%), with 95% confidence intervals in parentheses

*PPV* positive predictive value, *NPV* negative predictive value

**Fig. 4:**
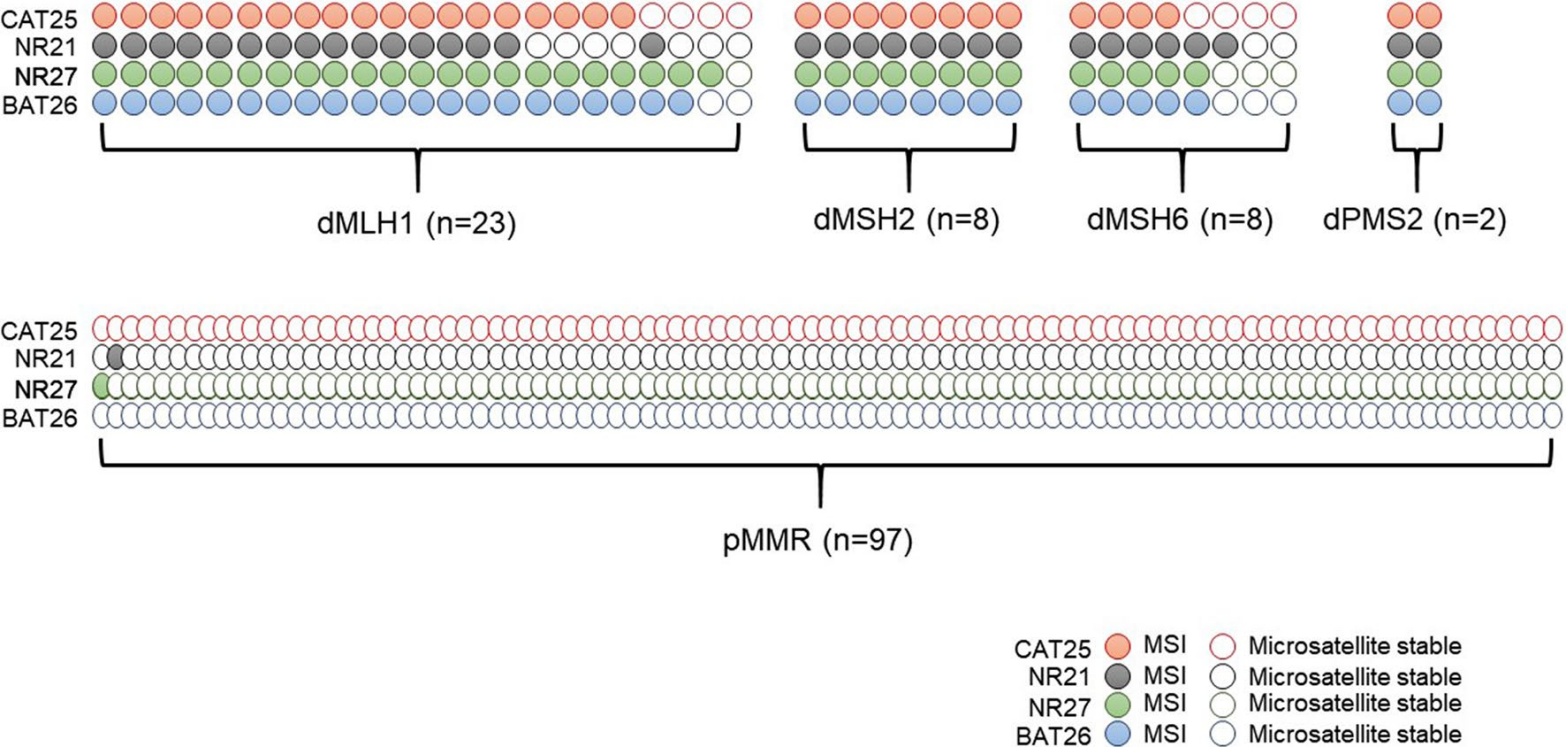
The performance of the Tetraplex system on the validation set. A colored circle denotes a positive for allelic variation (or MSI), and an empty circle indicates a negative for variation (or microsatellite stable) in this specific allele

The correlation of MSI data with MMR-protein-expression status demonstrated a sensitivity for dMLH1 (95.7%), dMSH2 (100%), dMSH6 (75.0%), and PMS2-deficient (100%) tumors using the Tetraplex system (Fig. 4).

## Discussion

Colorectal cancer (CRC) is believed to initiate as a benign adenomatous polyp, which subsequently develops into an advanced adenoma with high-grade dysplasia, and finally progresses to an invasive cancer. The clinical challenge remains a better understanding of the molecular basis of an individual’s susceptibility for developing CRC, and to determine factors that initiate development of tumor, drive its progression, and determine its responsiveness to antitumor agents. By Through the efforts of The Cancer Genome Atlas (TCGA), CRCs can now at least be classified into the two clusters; a hypermutator and a non-hypermutator phenotype [19, 20]. The hypermutated CRCs are also categorized into the following two subsets; tumors lacking DNA repair due to the mutations in the exonuclease domain of *DNA polymerase E* (*POLE*) characterized by an ultramutator phenotype, and a larger proportion of tumors with DNA mismatch repair deficiency (dMMR) and ensuing microsatellite instability (MSI) phenotype. This alterations in the *POLE* gene are distinct from the better-known dMMR which results in the classic MSI phenotype [20].

Recently, several studies have demonstrated that MSI is a positive predictor for immune-checkpoint blockade [9–11]. Hence, MSI analysis is now becoming important not only for the screening of Lynch Syndrome patients, but has a much larger role in identifying tumors exhibiting such a hypermutator phenotype that might respond to immune-checkpoint drugs. This includes analysis of sporadic as well as hereditary cases, of cancers with defects in the MMR system. The availability of a robust MSI assay that is fast, cost-efficient, and highly accurate for identifying dMMR in solid tumors is critical for its successful application in the clinic and research. In this study, we describe the development and application of a rapid and accurate MSI assay, which uses a single PCR reaction for the amplification of four mononucleotide microsatellite markers. This assay was subsequently validated for its usefulness in identifying MSI status in a series of pMMR and dMMR CRCs, as well as in ECs.

Standard MSI analysis using an NCI panel consisting of five microsatellite markers (two mononucleotide and three dinucleotide repeats) remains the preferred method in most clinical and research laboratories [14]. This is unfortunate, given that multiple studies have repeatedly shown that dinucleotide repeats are better suited to detecting MSI-low tumors, of which most are pMMR [14, 15], whereas mononucleotide MSI markers offer higher accuracy for detecting dMMR tumors [16].

We previously illustrated the usefulness of a pentaplex PCR system consisting of five mononucleotide markers, BAT25, BAT26, NR21, NR24, and NR27 [17]. A pairwise correlation and hierarchical-clustering analysis in this study clearly showed the weakest predictive values associated with NR24 and BAT25 as compared to the remaining three markers (BAT26, NR21, and NR27). Our observation of high sensitivity and positive predictive value with the reduced panel of three markers (BAT26, NR21, and NR27) versus the use of a panel consisting of all five markers has economic implications for MSI-based assays, as the use of this smaller marker panel might result in lower CRC-screening costs in the future [17]. One of the limitations of the NCI-panel markers is their inability to identify dMSH6 CRCs. Unfortunately, our previous study indicated that, even with the use of mono-markers in the pentaplex panel, the sensitivity of the assay to detect dMSH6 CRCs remained relatively low relative to dMMR due to the loss of other MMR proteins in CRCs [17]. This is significant, because the MutSα, a heterodimeric complex consisting of MSH2 and MSH6, preferentially recognizes base/base mismatches, as well as small insertion/deletion loops, containing one or two unpaired nucleotides in the DNA sequence, a subsequently participates in the repair of these lesions [21]. Therefore, one would expect that the functional loss of MutSα due to dMSH6 would lead to preferential instability in loci containing mononucleotide repeats [22]. Therefore, in this study, we included another mononucleotide marker, CAT25, to improve the sensitivity of our assay for identifying dMSH6 tumors [23]. In the CRC-validation set, our new four-marker panel detected dMSH6 tumors with a sensitivity of 86.7% (13 of 15 dMSH6 CRCs). This level of accuracy was also observed in the EC test set, where the system detected dMSH6 tumors with a sensitivity of 75.0% (6 of 8 dMSH6 ECs).

During the development of an assay exhibiting higher sensitivity for dMMR tumors, we considered that the most important factor for developing a more sensitive MSH6 marker would be the QMVR range. Indeed, the normal QMVR for the four markers used in this study was three to four bp. This robust range of QMVRs might improve the sensitivity for dMMR tumors, especially dMSH6 tumors. With regards to potential limitations, in our study the test set did not include any PMS2-deficient tumors. In addition, the sensitivity of this assay for detecting MSH6-deficiency is still under 90% in the both test and validation set.

## Conclusion

We describe a novel optimized PCR-based assay for screening MSI-positive solid tumors. This Tetraplex assay represents a simple and rapid screening approach with high-throughput capability, does not require amplification of matched normal DNA from a cancer patient, and exhibits a high degree of sensitivity and specificity for the detection of dMMR in ECs, as well as CRCs. We propose that this assay will help replace existing methodologies to aid in the improvement of detection of tumors with dMMR.

## Additional files

**Additional file 1: Table S1.** Primers for tetra-mononucleotide repeat PCR of tumors (Tetraplex) system.

**Additional file 2: Figure S1.** An example of MSI by Tetraplex PCR assay. Each arrow denotes alleles showing MSI. For each marker, gray shading indicates the adjusted QMVR.
