# Supplementary material for: Accuracy of four mononucleotide-repeat markers for the identification of DNA mismatch-repair deficiency in solid tumors
Source: J Transl Med. 2018 Jan 12;16:5. doi: 10.1186/s12967-017-1376-4 (PMC5767035; doi:10.1186/s12967-017-1376-4)
Supplement: Supplementary file 1 — Additional file 1: Table S1. Primers for tetra-mononucleotide repeat PCR of tumors (Tetraplex) system. [file 12967_2017_1376_MOESM1_ESM.docx]

**Table S1: Primers for tetra-mononucleotide repeat PCR of tumors (Tetraplex) system**

| **Name** | **Primer sequence- forward** | **Primer sequence- reverse** | **Size** |
| --- | --- | --- | --- |
| CAT25 | CTTCCCAACTTCCCTGTTCTTT | TGAGCTGAGATCGTGCCACT | 109 bp |
| NR21 | AGTCGCTGGCACAGTTCTATTT | ATTCCTACTCCGCATTCACACT | 133 bp |
| NR27 | AACCATGCTTGCAAACCACT | GCAGAGACCTTGTCAAAATTCA | 159 bp |
| BAT26 | CTGCGGTAATCAAGTTTTTAG | AACCATTCAACATTTTTAACCC | 182 bp |
