# Supplementary figures and images for: Accuracy of four mononucleotide-repeat markers for the identification of DNA mismatch-repair deficiency in solid tumors
Source: J Transl Med. 2018 Jan 12;16:5. doi: 10.1186/s12967-017-1376-4 (PMC5767035; doi:10.1186/s12967-017-1376-4)

## Slide 1
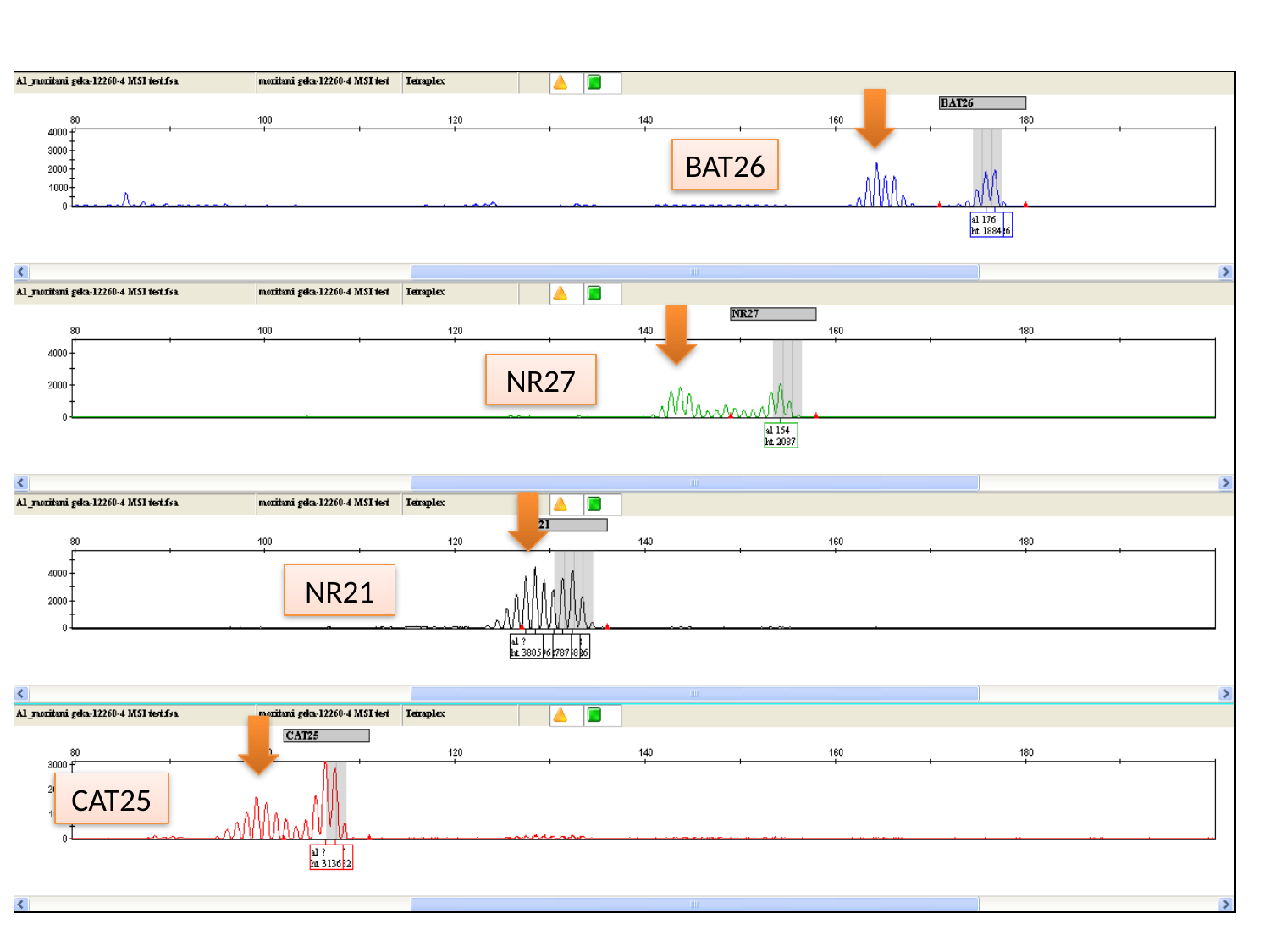

BAT26
NR27
NR21
CAT25

Supplement: Supplementary file 2 — Additional file 2: Figure S1. An example of MSI by Tetraplex PCR assay. Each arrow denotes alleles showing MSI. For each marker, gray shading indicates the adjusted QMVR. [file 12967_2017_1376_MOESM2_ESM.pptx]
